# Another “D” in MUDPILES? A Review of Diet-Associated Nondiabetic Ketoacidosis

**DOI:** 10.1177/2324709618796261

**Published:** 2018-08-23

**Authors:** Waqas Ullah, Mohsin Hamid, Hafez Mohammad Ammar Abdullah, Mamoon Ur Rashid, Faisal Inayat

**Affiliations:** 1 Abington Memorial Hospital, Abington, PA, USA; 2 University of South Dakota, Vermillion, SD, USA; 3 Florida Hospital, Orlando, FL, USA; 4 Jinnah Hospital, Allama Iqbal Medical College, Lahore, Pakistan

**Keywords:** Diet, Ketoacidosis, NDKA

## Abstract

Ketogenic diet or very-low-carbohydrate diet gained widespread popularity in the 1990s due to their favorable effects on weight loss and diabetes among others with good short-term safety data. People on ketogenic diets exist in a state of “dietary ketosis” in which the body production of ketone is equal to consumption and no harmful effects of ketonemia occur. However, in face of stress, the harmless “dietary ketosis” can lead to profound acid-base disturbances due to massive overproduction of ketone bodies that overwhelms the acid buffer system of the body. A handful of case reports have been published on this topic calling the safety of ketogenic diet into question. In this article, we chronicle a unique case of ketogenic (Atkins) diet–associated ketoacidosis, and we present a comprehensive literature review on the etiology of ketoacidosis.

## Introduction

Ketone bodies are formed by the deamination of amino acids and degradation of fatty acids in times of severe stress, diabetes, alcoholism, starvation, and pregnancy.^1^ Rarely, a low-carbohydrate ketogenic diet can also give rise to ketoacidosis.^2^ We present a case of a young nonpregnant female with no history of alcoholism, diabetes, or starvation presenting with dialysis-refractory metabolic acidosis due to Atkins diet–associated ketoacidosis. Urgent intubation, intensive care, and aggressive fluid resuscitation with insulin therapy led to the successful recovery of the patient. We also performed an extensive literature search and highlighted all reported literature of nondiabetic ketoacidosis (NDKA).

## Case Presentation

A 33-year-old Caucasian female was brought to the emergency department with possible syncope following lethargy and extreme exhaustion. Her mother found her on the floor of the restroom after hearing her falling down. According to her parents, she had mild flu-like symptoms, low-grade fever, and multiple episodes of nonbilious vomiting for 3 days before presentation. Her parents denied her ingestion of any medications or toxic substances intentionally or accidentally, and she did not have a past history of suicide attempts or ideation. Past medical history was only significant for high-functioning autism; she worked as a cashier at a fast food restaurant and was living with her parents. Her medications included methylphenidate and sertraline for years without any recent changes.

On presentation, her vitals included temperature 98.2°F, blood pressure 140/71 mm Hg, heart rate 136 beats per minute, respiratory rate 38/min, and oxygen saturation of 96% on ambient air. Examination revealed a Glasgow Coma Scale score of 10/15; mucous membranes were dry, and skin was cold to touch with decreased turgor. Breathing was deep and labored, chest was otherwise clear to auscultation; gastrointestinal and cardiovascular examinations were unremarkable.

## Investigations

The laboratory values are shown in Table 1.

**Table 1.** Laboratory Studies of Our Patient While She Was in the Intensive Care Unit.

Complete blood count	
Hemoglobin (g/dL)	12.2 (12-16)
White blood cells (/cmm)	34 800 (4000-12 000)
Platelet count (/cmm)	578 000 (140 000-400 000)
Differential type	
Neutrophils (%)	91
Lymphocytes (%)	2
Monocytes (%)	7
Chemistry	
Glucose (mg/dL)	126 (70-100)
Serum osmolarity (mOsm/kg)	323 (275-295)
Sodium (mmol/L)	141 (135-145)
Potassium (mmol/L)	5.7 (3.5-5.1)
Chloride (mmol/L)	111 (98-110)
Blood urea nitrogen (mg/dL)	17 (<23)
Creatinine (mg/dL)	1.32 (<1.11)
Bicarbonate (mmol/L)	<5 (20-31)
AST (U/L)	21 (4-34)
ALT (U/L)	20 (<55)
ALP (U/L)	130 (40-150)
Total bilirubin (mg/dL)	0.2 (0.2-1.2)
HbA1c (mg/dL)	5.6
Serum lactate (mEq/L)	1.1 (normal <2.0)
Anion gap	27 (8-12)
Arterial blood gas	
pH	6.8 (7.35-7.45)
pCO_2_ (mm Hg)	16.9 (35-45)
pO_2_ (mm Hg)	120 (80-100)
HCO_3_ (mmol/L)	2.6 (21-29)
SaO_2_ (%)	97.4 (95-100)

Abbreviations: AST, aspartate aminotransferase; ALT, alanine aminotransferase; ALP, alkaline phosphatase; HbA1c, hemoglobin A1c; pCO_2_, partial pressure of carbon dioxide; pO_2_, partial pressure of oxygen; HCO_3_, bicarbonate; SaO_2_, oxygen saturation.

Serum and urine toxicology was negative for alcohol, salicylate, acetaminophen, and other toxic alcohols. Urine pregnancy test was negative; however, urinalysis was positive for ketones (+4). Computed tomography scan of head and chest X-ray were unremarkable.

## Differential Diagnosis

Initial suspicion of the cerebrovascular accident was ruled out by the normal computed tomography scan of the head. Sepsis, diabetic ketoacidosis, poisoning with toxic alcohols, and pregnancy-induced ketosis were considered, but they were ruled out with the appropriate studies.

## Treatment

She was initially stabilized with aggressive volume resuscitation, sodium bicarbonate therapy, and ventilator support. Broad-spectrum antibiotics (vancomycin and piperacillin/ticarcillin) were started due to the possibility of sepsis from unknown origin. Persistent metabolic acidosis in spite of bicarbonate therapy led to one session of hemodialysis that improved serum bicarbonate levels to 16 mEq/L from 2.5 mmol/L. After a few hours of hemodialysis, repeat laboratory tests showed worsening acidosis with serum bicarbonate dipping down to 12 mmol/L. At this point, endocrinology was consulted and serum was tested for possible ketonemia, which turned out to have β-hydroxybutyrate (β-hB) levels of 120.1 mg/dL (normally less than 3 mg/dL). On further questioning, her parents mentioned that she was on a “weight-loss diet” for about 2 months. Suspecting the diagnosis of ketogenic diet–induced ketoacidosis, she was started on intravenous (IV) insulin along with dextrose 10%.

## Outcome and Follow-up

She improved rapidly after the start of IV insulin and dextrose infusions. Metabolic acidosis and anion gap (AG) resolved within a day and β-hB levels normalized. She was then extubated and shifted to a general medicine floor on the second day of her presentation. On further questioning, she admitted to following a strict commercially available ketogenic diet (Atkins diet) for the past 2 months. She was discharged home in a stable condition on day 5 of hospitalization with the diagnosis of the ketogenic diet (Atkins diet)–induced euglycemic metabolic ketoacidosis. A follow-up after 3 months revealed that she was doing well and was not following the Atkins diet anymore.

## Discussion

Over the past century, numerous different diets have been studied and tried to help weight loss. The 2 basic diets popularized are the low-fat and the low-carbohydrate diet (LCD). The LCD has increased popularity in the latter part of the past century, especially after the introduction of Atkins diet by R. C. Atkins in 1972.^3^ Atkins diet is a special type of LCD, which is protein rich and has high-fat contents. The beneficial effect of LCD in an Atkins regime is believed to be due to the use of higher energy ketones rather than glucose for the production of energy. Even though studies have shown that LCD is effective for weight loss and causes improvement in cardiovascular risk factors, there are still controversies surrounding the use of this diet, one of the most serious of which is NDKA.^4^

NDKA is the presence of ketoacidosis in the absence of diabetes and is typically associated with starvation, alcoholism, hyperthyroidism, and lactation. However, this can also occur rarely with LCD, especially in patients with comorbid conditions and intercurrent illness. Our case is unique in the sense that it occurred in an otherwise healthy young female with no significant comorbidities.

**Figure 1.**
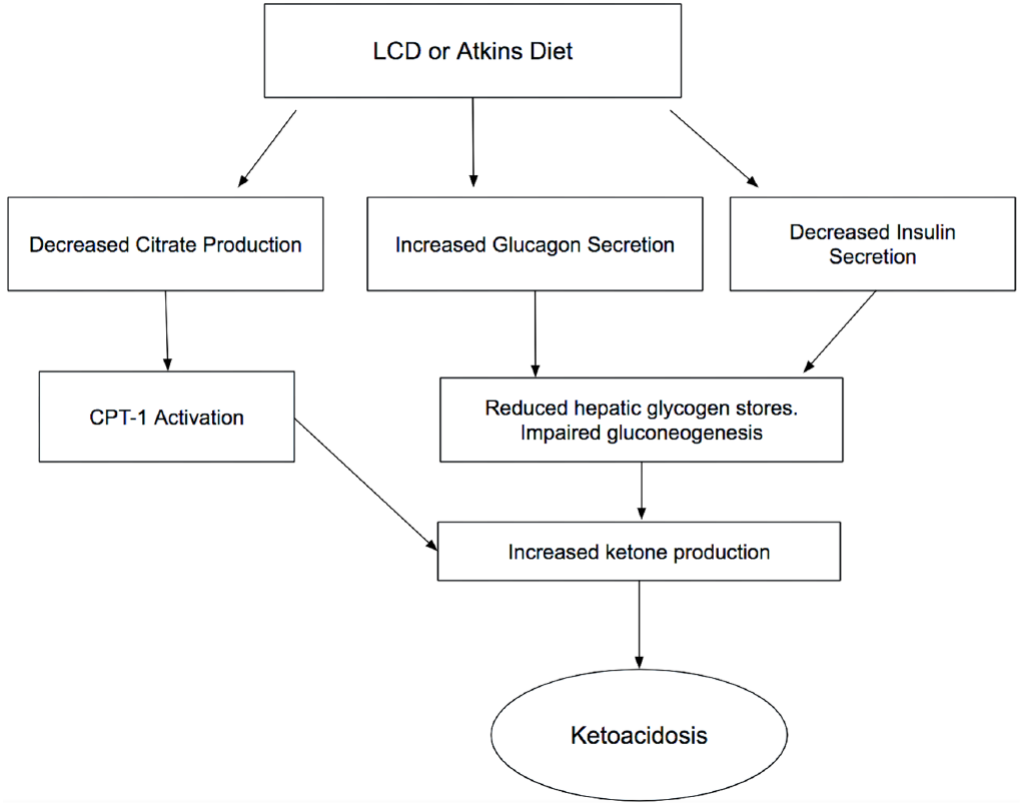
Flow sheet of the pathophysiology of nondiabetic ketoacidosis.

There are a few mechanisms by which an LCD induces ketosis. Normally in high-carbohydrate states, aerobic glycolysis produces citrate as one of the intermediate metabolites. This citrate is a signal for a high-glucose state and inhibits the activity of carnitine palmitoyltransferase complex-1 (CPT-1).^2^ CPT-1 complex is responsible for the β-oxidation of fatty acids, and its inhibition prevents the metabolism of fatty acids and the production of ketones in carbohydrate-rich states. The opposite occurs in low-carbohydrate states. With no citrate, the CPT-1 is activated and extensive β-oxidation of fatty acids occurs. The liver then uses this energy to make ketone bodies, which provide energy to important organs, including the brain, heart, and kidneys.^2^ Moreover, fat-rich diet and LCD can enhance glucagon secretion and lower insulin secretion, and a high glucagon-to-insulin ratio also promotes ketosis.^5,6^ Excessive accumulation of ketone bodies in the body occurs when its production is greater than consumption, leading to ketoacidosis. We believe that the low-carbohydrate and high-plasma fatty acid concentrations, compounded by the absence of carbohydrate-induced inhibition of β-oxidation of fatty acids might be the cause of NDKA in patients on an Atkins diet. The pathophysiology of LCD or Atkins diet-induced ketoacidosis is shown in Figure 1.

A comprehensive literature search using the MEDLINE databases (PubMed and Ovid) showed only a handful of cases on ketoacidosis in nondiabetic patients. Different Medical Subject Headings (MeSH) like “non-diabetic,” “euglycemic,” “normoglycemic,” “normal sugar levels,” “ketoacidosis,” “ketonuria,” “ketonemia,” “ketosis,” “metabolic acidosis,” “fatty acids,” “metabolites,” “acetoacetate,” and “beta-hydroxybutyrate” were combined using Boolean operators. A total of 127 cases were initially obtained; after removing 36 duplicate studies, 93 studies were read in full-text form. Twenty-seven out of 93 cases were found relevant to our study, but only 22 cases had sufficient data for extraction.^2,5-25^

**Figure 2.**
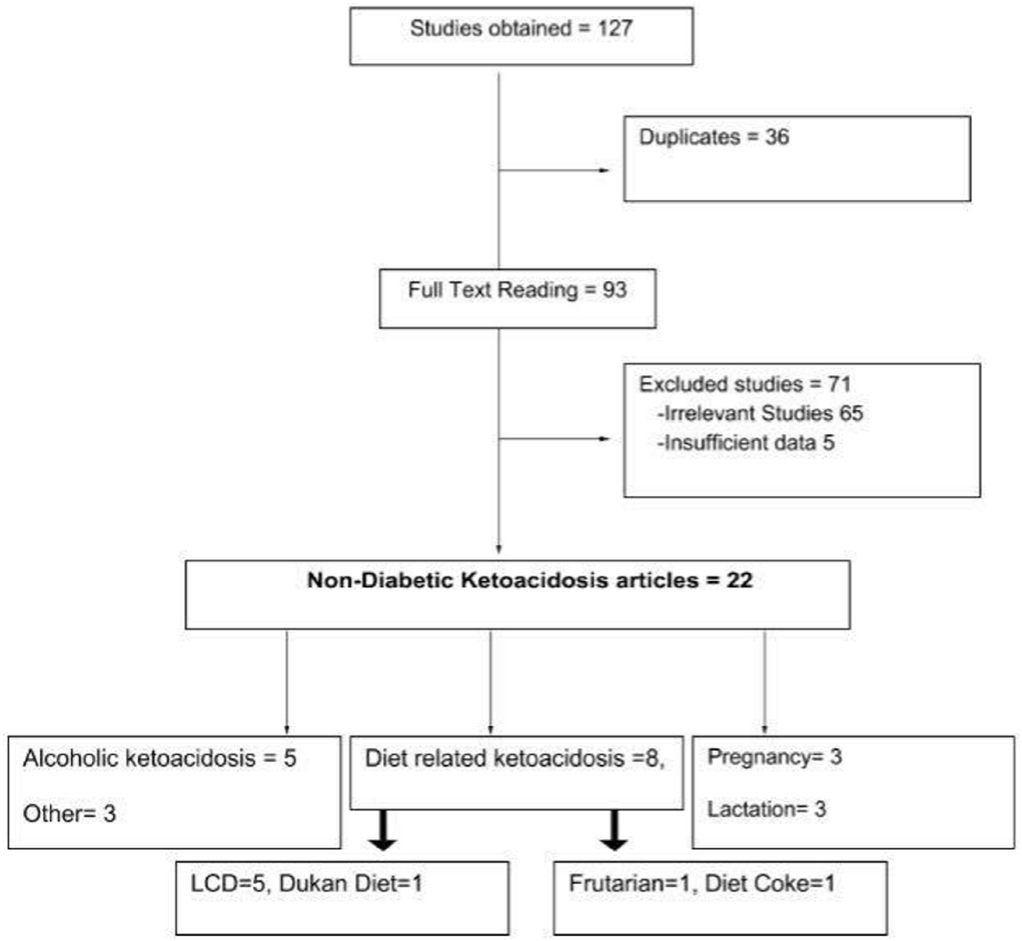
Flow sheet of studies on nondiabetic ketoacidosis.

Further stratification of the selected cases showed that NDKA was most commonly reported in chronic alcoholics (5 case reports including 9 patients) and LCD users (5 cases).^2,6,7,9,11,17,21^ Only 3 cases were reported in pregnancy and lactation each.^12,13,15^ It is interesting to note that the pregnant and lactating patients had overriding starvation or low-carbohydrate intake as precipitating factors for ketoacidosis. There was only a single case of euglycemic ketoacidosis reported with diets like Dukan diet, diet coke, and fruitarian diet each and a single case each with hyperthyroidism, bariatric surgery, and salicylate use^22,24^ (Figure 2). Most of these patients had an underlying condition like pregnancy, asthma, pneumonia, or hyperthyroidism, which might have made them vulnerable to develop ketoacidosis. Our patient is unique as she was a previously healthy individual who presented as syncope mimicking cerebrovascular accident. The underlying cause was the Atkins diet, which has never been reported before as the cause of severe NDKA.

NDKA was most commonly reported in female patients (n = 16) when compared with male patients (n = 11). The age distribution ranged from 5 to 79 years. More than 80% of the patients were at the extreme of ages and with other comorbid conditions signifying that stressful or lower metabolic reserve makes patients susceptible to NDKA. Our patient, however, was unique that flu was the only association, and she was relatively younger than the majority of reported patients. Most patients presented with gastrointestinal symptoms like nausea, vomiting, and diarrhea (n = 13) or mild central symptoms like a headache, dizziness, and confusion (n = 5). Cardiovascular symptoms like palpitations (n = 3) were also noted in a few cases; our patient surprisingly presented with syncope with a low Glasgow Coma Scale score of 10/15 and respiratory failure. Almost all patients were normotensive and were maintaining oxygen saturation at room air. All of them were found to have positive urinary ketones or serum ketonemia in association with high AG metabolic acidosis in the absence of hyperglycemia and a history of diabetes. This was confirmed by arterial blood gases, serum and urinary ketones, and an HbA1c. The β-hB was the most commonly used tested ketone body with a value ranging from 80 till 9000 µmol/L in the previously reported cases; in our case, the value was above 10 000 μmol/L. High AG metabolic acidosis in reported cases was spread between a pH of 6.8 to 7.2, HCO_3_ 2.9 to 10 mEq/L, and AG 21 to 28. In our patient, however, the pH was very low 6.6, HCO_3_ of 2.6, and AG 30. These findings were alarming for us and are important for readers suspecting that Atkins diet is associated with serious NDKA in terms of its presentation and laboratory findings. It also signifies that a ketogenic diet can cause severe symptoms and complications like syncope and respiratory failure even in a previously healthy young patient when they encounter a stressful condition like seasonal flu. The characteristics of previously reported cases are summarized in Table 2.

**Table 2.** Previously Reported Cases of Nondiabetic Euglycemic Ketoacidosis^a^.

No.	Author/Reference	Age/Gender	Presentation	BMI, T, BP, HR, RR, SaO_2_	Laboratory Studies	Diagnosis	Associations/Comorbidities	Management	Outcome	Follow-up
1	Shah and Isley^2^	51/Female	Vomiting	BMI 21.7	Anion gap acidosis, ketonuria, normal HbA1c	HAGMA and urinary ketones	Low-carb for 4 years (20 g/day)	IV fluids and insulin	Improved and discharged	NA
2	Hudak et al^5^	32/Female	Tachypnea, tachycardia	NA	Urine acetone +3, Hb 14.5, WBC 10 200. RBS 108, pH 6.8, HCO_3_ 3, PCO_2_ 8, AG 28, lactate 1	HAGMA and urinary acetone	Fasting and lactation	ICU care: IV normal saline, sodium bicarbonate (8.4%), potassium, phosphate, and glucose	Improved and discharged on day 10	NA
3	von Geijer and Ekelund^6^	32/Female	Nausea/vomiting, palpitations, trembling, extremity spasms	T36; BP 110/80; HR 102; RR 12; SaO_2_ 96%	Ketones 7.1, pH 7.20, pCO_2_ 25, RBS 103, lactate 1.0	HAGMA and ketosis	LCHF diet (carb <20 g/day)	IV glucose infusion, IV insulin	Improved and discharged on day 3	1 month, stable
4	Iwata et al^7^	76/Female	Dizziness, headache	BMI 22.8; T36; BP 106/94; HR 100; RR 15; SaO_2_ 99%	β-hB 6663, Hb 15.6, BUN 26.3,WBC 12 500, lactate 27.2, pH 7.28, AG 27.3, RBS 93, HbA1c 6.8%	HAGMA and ketosis	Hamburger steak only(carb = 12.7 g/day)	IV normal saline and regular diet	Improved and discharged on day 3	2 months, stable
5	Lewis^8^	5/Male	Vomiting/diarrhea, fever	T36.8; BP 110/70; HR 100; RR 20	Serum ketone 80, Hb 13.5, HCO_3_ 6, RBS 82, AG 27, pH 7.14, pCO_2_ 14, lactate 0.7	HAGMA and serum ketones	Diet of aspartame-sweetened 7-UP	IV dextrose in water with 0.45% NaCl and potassium acetate	Improved and discharged on day 2	NA
6	Freeman et al^9^	42/Female	Nausea/vomiting	BMI 25.6; T36; BP 114/65; HR 76; RR 18; SaO_2_ 100%	WBC 16 900, RBS 99, pH 7.21, HCO_3_ 10, lactate 0.9, urine ketone >150	HAGMA and urinary ketones	Dukan diet (low-carb, high-protein), fasting	IV 2 L of normal saline, ondansetron, and promethazine	Improved and discharged on day 2	NA
7	Dahl et al^10^	24/Male	Malaise, vomiting/dyspnea	HR 140; RR 60	pH 7.08, AG 36, Hb 14.3, WBC 19 600, RBS 145, lactate 0.9, urine ketones +4, serum acetoacetate 4.5	HAGMA, serum acetoacetate and urinary ketones	Water and Diet Coke only, herpes stomatitis	IV normal saline and sodium bicarbonate	Improved and discharged	None
8	Causso et al^11^	35/Male	Aggressiveness, incoherent speech, fasting for over a week	BMI 16	RBS 57, pH 7.08, HCO_3_ 7.4, pCO_2_ 25, Hb 13.7, lactate 2.6, acetoacetate 5.1, urine ketone >150	HAGMA, serum acetoacetate, and urinary ketones	Fruitarian (ate only fruits for 10 years) due to undetermined psychotic disorder	IV normal saline, IV dextrose, bicarbonate	Improved and discharged	None
9	Monnier et al^12^	29/Female	Dyspnea/anorexia, fatigue, weight loss	NA	pH 7.11, Hb 11, WBC 10 000, PaCO_2_ 8, PaO_2_ 136 mm Hg, HCO_3_ 2.9	HAGMA and ketosis	Restricted diet, lactation, and gastric bariatric surgery	IV normal saline	Improved and discharged in 7 hours	NA
10	Wuopio et al^13^	21/Female	Nausea/dyspnea, headache	NA	pH 6.9, RBS 120, B-acetones 10, AG 21	HAGMA and serum acetone	Low-carbohydrate diet and lactation	IV Ringer lactate and IV glucose	Improved and discharged on day 1	NA
11	Milroy and Parai^14^	3/Female	Death	NA	β-hB 3966, RBS 89, HbA1c 4.7%	HAGMA and ketosis	Starvation, pneumonia, and Armanni-Ebstein lesion	NA	NA	NA
12	Wei et al^15^	36/Female	Dyspnea/vomiting/anorexia	BMI 26.5; T37; BP 126/78; HR 138; RR 25	β-hB 7216, WBC 15 850, RBS 152, HbA1c 4.8%, pH 7.21, HCO_3_ 11, PCO_2_ 28, AG 15, lactate normal	HAGMA and ketosis	Starvation, pregnancy, asthma	ICU care: intubation, albuterol, dexamethasone, dextrose in normal saline and C-section	Improved and discharged on day 14	NA
13	Scholte and Boer^16^	26/Female	Dyspnea/vomiting and abdominal discomfort	BMI 44.3; T37; BP 150/90	Urine ketone 7.8, HbA1c 5.4%, pH 7.30, HCO_3_ 9, AG 21, RBS 120	HAGMA and urinary ketones	Starvation, pregnancy, hemochromatosis	ICU care: IV antiemetics and normal saline, 8.4% sodium bicarbonate	Improved and discharged on day 5	NA
14	Jain et al^17^	50/Female	Comatose state, vomiting/anorexia	T37; BP 110/80; HR 120; RR 34; SaO_2_ 93%	β-hB 14 860, RBS <20 mg/dL, HCO_3_ 9, AG 9, pH 7.2, pCO_2_ 24, lactate 2.3, ketonuria	HAGMA urine and serum ketones	Chronic alcoholism and chronic liver disease	ICU intubate, IV normal saline and 5% dextrose with thiamine and folate	Vegetative state	10 months
15	Gill and Yong^18^	39/Male	Abdominal pain and vomiting	HR 120	Ketonuria, WBC 11 500, pH 7.18, pCO_2_ 20, pO_2_ 94, HCO_3_ 10	HAGMA and urine ketones	Chronic alcoholism and pancreatitis	IV 0.9% NaCl infusion and glucose, potassium, insulin infusion	Improved and discharged on day 3	6 months, third episode
16	Devenyi^19^	71/Female	Vomiting/fasting	NA	Raised serum β-hB and ketonuria, RBS 31, pH 6.9	HAGMA urine and serum ketones	Chronic alcoholism	IV hypertonic dextrose	Improved twice	Died, third episode
17	Bernuau et al^20^	N/A	Vomiting/starvation	NA	Raised serum β-hB and ketonuria, AG 25 to 41, lactate 0.9	HAGMA urine and serum ketones	Chronic alcoholism	IV fluids	NA	NA
18	Platia and Hsu^21^	5 patients, 28-39/Male	Vomiting/stuporous, comatose	NA	Raised β-hB up to 9800, low glucose level	HAGMA urine and serum ketones	Chronic alcoholism	IV thiamine, glucose, and dextrose	Improved and discharged on day 2	1 and 3 months, stable
19	Arena et al^22^	78/Female	Confusion and lethargy	T 37.4; BP 140/72; HR 84; RR 28	Raised serum ketones, salicylate 60.8 mg/dL, RBS 38, pH 7.28	HAGMA and serum ketones	Salicylates for degenerative joint disease	IV dextrose and sodium bicarbonate	Improved and discharged	NA
20	Wood and Kinlaw^23^	41/Female	Vomiting/diarrhea, palpitation, and weight loss	T 27.3; BP 146/75; HR 112	β-hB 5210, pH 7.36, pCO_2_ 26, HCO_3_ 14.5, RBS normal	HAGMA and serum ketones	Hyperthyroidism	IV normal saline, propranolol, propylthiouracil, thiamine	Improved and discharged	NA
21	Vestergaard et al^24^	79/Female	Stroke and weight loss ~13 kg	NA	NA	Metabolic acidosis with elevated poly-3-hydroxybutyrate	Pneumonia and dehydration	IV normal saline, potassium, bicarbonate, and insulin	Improved and discharged on day 14	NA
22	Valkenborgh and Bral^25^	19/Female	Metabolic acidosis after bariatric surgery	BMI 40.5; T 36; BP 106/94; HR 100; RR 16; SaO_2_ 92%	pH 7.28, pCO_2_ 34, HCO_3_ 16	Starvation-induced ketoacidosis	Bariatric surgery	Oral glucose therapy	Improved and discharged on day 3	NA
23	The present report	33/Female	Syncope, respiratory failure	BMI 27; T 37; BP 140/71; HR 136; RR 22; SaO_2_ 96%	β-hB 10 600, pH 6.6, pCO_2_ 16.9, pO_2_ 120.5, HCO_3_ 2.6, AG 30, RBS 126, HbA1c 5.1%	HAGMA and serum ketones	Atkins diet and flu	ICU: intubation, hemodialysis and IV dextrose saline and insulin	Improved and discharged on day 7	3 months, stable

Abbreviations. BMI, body mass index; HbA1c, hemoglobin A1c; HAGMA, high anion gap metabolic acidosis; IV, intravenous; NA, not available; WBC, white blood cells; RBS, random blood sugar; HCO_3_, bicarbonate; pCO_2_, partial pressure of carbon dioxide; AG, anion gap; ICU, intensive care unit; BP, blood pressure; HR, heart rate; RR, respiratory rate; SaO_2_, oxygen saturation; LCHF, low-carb high fat; β-hB, β-hydroxybutyrate; Hb, hemoglobin; BUN, blood urea nitrogen; NaCl, sodium chloride; pO_2_, partial pressure of oxygen.

a Units of measurement: BMI in kg/m^2^; Temperature (T) in Celsius; BP in mm Hg; HR is in beats per minute; RR is in breaths per minute; SaO_2_ is in percentage; β-hB is in µmol/L; acetoacetate is in mmol/L; Hb, RBS, lactate, and BUN are in mg/dL; WBC is in counts/µL; AG is in mEq/L; HCO_3_ is in mmol/L; pCO_2_ is in mm Hg; and β-acetones in 10 mmol/L.

NKDA is usually managed with IV dextrose along with insulin, IV bicarbonate, correction of electrolyte imbalances, and supportive treatment until the acidosis is resolved. Though NDKA is a serious and potentially life-threatening condition, timely diagnosis and proper management as in our case leads to good outcomes. Outcomes were not reported in all the cases; however, in cases where details were available, all the patients recovered with no significant long-term sequelae.

## Conclusions

- Starvation, pregnancy, lactation, and ketogenic (Atkins) diet–associated ketoacidosis can be severe and possibly fatal if not treated promptly.
- Physicians should consider diet-associated ketoacidosis in previously nondiabetic patients presenting with severe metabolic acidosis.
- Hemodialysis is only helpful as a temporizing measure but has no role as definitive management.
- Intravenous insulin is the mainstay of treatment even in patients with normal glucose levels; however, intravenous dextrose should be given to avoid hypoglycemia.
- To summarize, Atkins diet and other previously reported diets like Dukan, fruitarian, aspartame, and other low-carb high-fat diets should be considered as an extra “D” in the mnemonic “MUDPILES” for causes of high anion gap metabolic acidosis.
